# Avatrombopag increased platelet count in a patient with chronic immune thrombocytopenia refractory to multiple lines of treatment

**DOI:** 10.1097/MBC.0000000000001232

**Published:** 2023-06-02

**Authors:** Weronika Lebowa, Joanna Zdziarska, Tomasz Sacha

**Affiliations:** a Department of Hematology, University Hospital, Jagiellonian University Medical College; b Jagiellonian University Medical College, Doctoral School of Medical and Health Sciences, Faculty of Medicine, Cracow, Poland

**Keywords:** autoimmune thrombocytopenia, avatrombopag, immune thrombocytopenia, thrombocytopenia

## Abstract

We present a case of a 30-year-old man suffering from chronic refractory immune thrombocytopenia (ITP) from early childhood. The patient was treated with all the therapeutic methods available in Poland, without platelet response: corticosteroids, intravenous immunoglobulins, splenectomy, cyclophosphamide, vinblastine, azathioprine, mycophenolate mofetil, rituximab, ciclosporin A, romiplostim, and eltrombopag. He continued to function persistently with deep thrombocytopenia, symptoms of hemorrhagic diathesis, and one episode of spontaneous subarachnoid bleeding. In April 2022, at the age of 29, the patient received avatrombopag. Within 4 weeks of starting avatrombopag 20 mg daily for 2 weeks and then 40 mg daily, he reached a platelet (PLT) count of 67 x 10^9^/l. In the next month, platelets fell below 30 x 10^9^/l, but subsequently the count increased to 47 x 10^9^/l, then to 52 x 10^9^/l, and remained stable. The symptoms of cutaneous hemorrhage diathesis have resolved completely since avatrombopag was introduced and did not reappear despite the decrease in PLT count.

## Introduction

Immune thrombocytopenia (ITP) is an acquired autoimmune disease characterized by a decrease in the peripheral platelet (PLT) count below 100 x 10^9^/l without any other explanation [1]. Treatment of ITP includes pharmacotherapy and splenectomy. First-line treatment is based on corticosteroids and intravenous immunoglobulins. Splenectomy, thrombopoietin receptor agonists (TPO-RAs), and rituximab are the principal components of second-line treatment. Other therapies, including fostamatinib, immunosuppressive agents, danazol, dapsone, and combinations, should be considered if second-line treatment was ineffective or cannot be used [2,3]. In 2009, an International Working Group defined refractory ITP as a disease that does not respond to or relapses after splenectomy and requires treatment to reduce the risk of clinically significant bleeding [4]. In recent years, broader definitions of refractory ITP have been proposed. One of them, except for patients who failed to or relapsed after splenectomy, also includes patients with refractoriness to maintenance therapy who were disqualified from splenectomy or did not accept surgery as an option, as well as patients in whom the primary objective of treatment is improvement in health-related quality of life, not only prevention of severe bleeding [5]. Another definition describes refractory ITP as a disease that does not respond to at least two treatment methods, the PLT count is consistently very low and accompanied by bleeding, and there is no single medication that increases PLT count [6]. Yet, another concept defines refractory patients as patients who failed two or more medical therapies preceded by treatment with corticosteroids or immunoglobulins regardless of splenectomy [7].

TPO-RAs (romiplostim, eltrombopag, and avatrombopag) stimulate the production of megakaryocytes and ultimately platelets in the bone marrow by binding to and activating the TPO receptor. The efficacy of eltrombopag and romiplostim in increasing PLT counts shown in clinical trials is in the range of approximately 80% [8–11]. For avatrombopag, clinical experience is limited, as the drug was approved for the treatment of chronic ITP by the U.S. Food and Drug Administration in 2019 [12]. There are no clear guidelines that define which TPO-RA should be administered first in the treatment of chronic ITP. In 2019, the American Society of Hematology suggested a choice between romiplostim or eltrombopag depending on the patient's preferences for the route of administration [13]. Although there are no recommendations for initiating TPO-RA treatment with avatrombopag, there are two retrospective observational studies that have shown that avatrombopag may be effective in maintaining platelet response in chronic ITP, even after the failure of treatment with romiplostim and eltrombopag [14,15], which is also shown in the presented case report.

This case is the first published in Poland description of a patient with chronic refractory ITP who was successfully treated with avatrombopag. Written informed consent was obtained from the patient for the publication of this case report.

## Case report

**Fig. 1:**
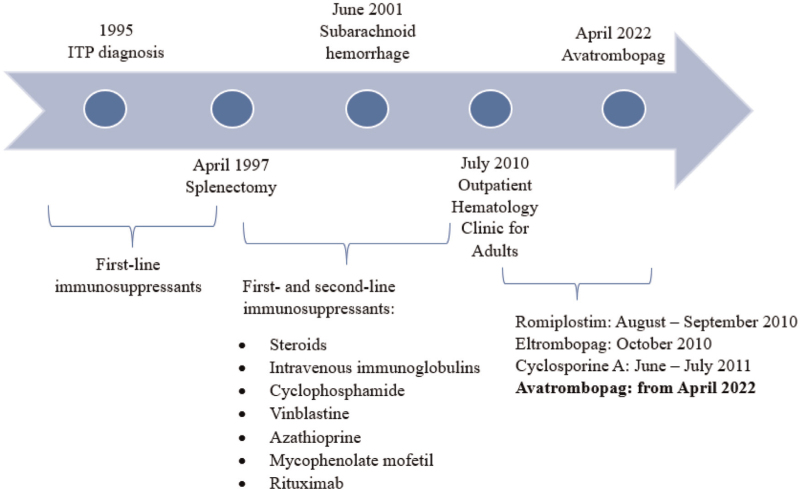
Treatment timeline.

We present a case of a 30-year-old male patient with chronic refractory ITP (Fig. 1). In 2010, the patient was referred to the Adult Outpatient Hematology Clinic at the University Hospital of Jagiellonian University Medical College in Krakow, Poland. He was diagnosed with ITP at the age of 3 when he presented with petechiae. His platelets were not measured until 3 years of age when the first symptoms of diathesis appeared. Congenital thrombocytopenia was ruled out based on a transient poststeroid platelet response. His medical history included chronic streptococcal tonsillitis treated periodically with antibiotics and anxiety-depressive syndrome. The patient's family history was unremarkable for thrombocytopenia and bleeding.

Before the age of 5, the patient was qualified for splenectomy after initial immunosuppressive treatment. Splenectomy was performed with no long-term therapeutic effect; thrombocytopenia relapsed within 6 months. The scintigraphy revealed no accessory or regenerative splenic tissue. To exclude secondary ITP, abdominal ultrasound, tests for hepatitis B virus, hepatitis C virus, HIV, cytomegalovirus, Epstein--Barr virus, *Helicobacter pylori*, autoantibody tests (antinuclear antibodies, antineutrophil cytoplasmic antibodies, antiphospholipid antibodies), and immunoglobulin levels were performed, which were all normal. Bone marrow biopsy revealed reactive stimulation of the megakaryocytic lineage (50–60 cells/μl, the normal age-adjusted range was up to 20 cells/μl) with the correct maturation pathway. No evidence of an underlying hematologic malignancy was revealed.

In the following years, all the treatment modalities available in Poland were exhausted; the patient was refractory to steroids (prednisone, methylprednisolone, and dexamethasone), immunoglobulins, cyclophosphamide, vinblastine, azathioprine, mycophenolate mofetil, and rituximab. The patient was chronically functioning with a PLT count ranging from 4 x 10^9^/l to 20 x 10^9^/l. The clinical presentation included primarily cutaneous hemorrhagic diathesis: petechiae, nose bleeding, and gingival bleeding. Due to multidrug resistance, the patient was left under observation without treatment. Rescue treatment with oral methylprednisolone was administered depending on the presence and severity of the symptoms. Steroids did not significantly increase PLT count but reduced the intensity of hemorrhagic diathesis.

At the age of 9, the patient experienced subarachnoid hemorrhage not preceded by trauma, with a PLT count of approximately 30 x 10^9^/l. He was hospitalized and treated conservatively. MRI of the head revealed the presence of vascular malformation. The bleeding did not leave any permanent neurological deficits.

**Fig. 2:**
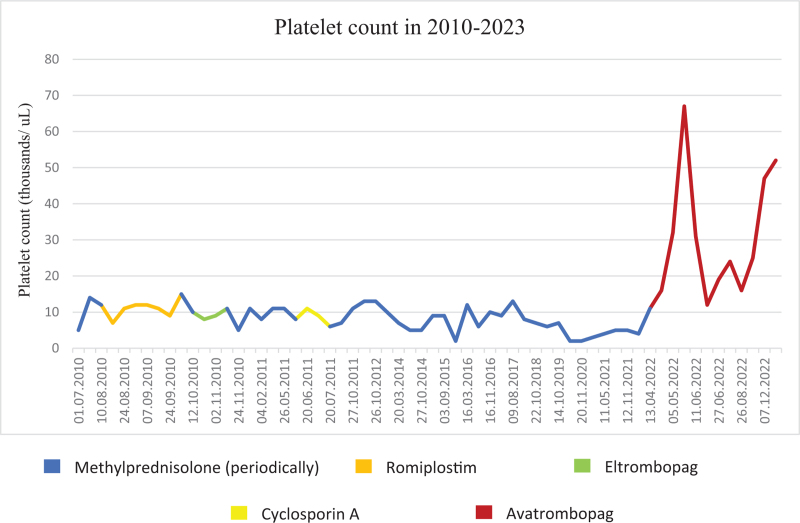
Platelet count in 2010–2023.

In 2010, after the admission of the patient to the Outpatient Adult Hematology Clinic of the University Hospital, Jagiellonian University Medical College, treatment with thrombopoietin receptor agonists was applied, first with romiplostim and after its failure with eltrombopag. Romiplostim was administered subcutaneously for 8 weeks (one injection per week) in increasing doses, from 1 μg/kg body weight to 8 μg/kg body weight. The patient was then switched to oral eltrombopag at an initial dose of 50 mg daily for 4 weeks. For both TPO-RAs, treatment was discontinued due to lack of efficacy (Fig. 2). In 2011, a 2-month therapy with cyclosporine A was introduced at a dose of 200 mg daily. However, the PLT count remained in the range of 10 x 10^9^/l. The periodic treatment cycles with oral methylprednisolone were reintroduced in the event of exacerbation of hemorrhagic diathesis usually due to infections. The patient required such treatment on average once a quarter. In addition, etamsylate was used as supportive therapy.

**Fig. 3:**
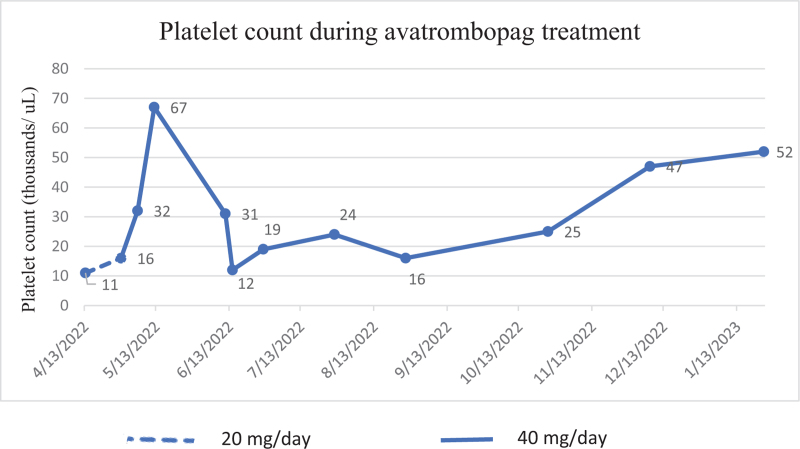
Platelet count during avatrombopag treatment.

In March 2022, an application for administering avatrombopag was prepared. Treatment with avatrombopag at a dose of 20 mg per day was initiated on April 14, 2022. After 2 weeks, the daily dose of the drug was increased to 40 mg. The treatment was well tolerated and effective. After 1 month, platelets increased to 67 x 10^9^/l (Fig. 3). The symptoms of cutaneous hemorrhage diathesis resolved completely since the introduction of avatrombopag and did not reappear despite the drop in the PLT count (25 x 10^9^/l). In the next follow-up, the PLT count increased to 47 x 10^9^/l, then to 52 x 10^9^/l, and remained stable since October 25, 2022. The patient was benefiting from treatment; therefore, therapy was continued. PLT counts and therapy over time are shown in Fig. 2.

## Discussion

Our patient who presented with ITP refractory to multiple treatment lines was finally successfully treated with avatrombopag. He was the first patient to switch to avatrombopag in our clinic. In Poland, avatrombopag is available for the treatment of refractory ITP since March 2022. This case study demonstrates that avatrombopag may be a favorable option in the treatment of chronic refractory ITP even after a prior failure of therapy with alternative TPO-RAs.

The first step in the management of refractory ITP is to reassess the diagnosis, as some of these patients have been misdiagnosed [5–7]. The proper diagnosis is complicated by the fact that there are no specific tests that confirm ITP. ITP is a diagnosis of exclusion of other known reasons for thrombocytopenia. The strongest evidence for the presence of ITP is a platelet response to standard therapy. In the absence of a response to treatment, an alternative diagnosis should be taken into account, including secondary ITP [6]. In our patient series of laboratory tests (as listed in the case presentation), bone marrow examination was performed to rule out hematological malignancy and secondary ITP.

The primary goal of ITP treatment is to protect patients from life-threatening bleeding by keeping the PLT count at the hemostatic level (> 20–30 x 10^9^/l in most cases) [3]. Patients with refractory ITP generally do not respond to multiple lines of treatment. Response to treatment is assessed based on PLT count combined with the clinical picture of the patient. Complete response (CR) is defined as an increase in PLT count at least 100 x 10^9^/l, while response means PLT count at least 30 x 10^9^/l and a doubling from baseline with no bleeding [16]. Lack of response is associated with an increased risk of bleeding, including life-threatening conditions, and a lower health-related quality of life. If a refractory ITP is identified, a careful individual approach is required. According to the guidelines, patients with a PLT count greater than 30 x 10^9^/l and without symptoms can be left untreated [16]. In some refractory patients, especially asymptomatic or minimally symptomatic, expectant attitude and observation without treatment may be considered as a balance between bleeding risk and treatment toxicity. This approach can be implemented in stable patients, even with a PLT count of less than 10 x 10^9^/l [7,17]. Many patients with refractory ITP (as in our presented case) can function satisfactorily with low PLT counts with only periodic courses of corticosteroids or antifibrinolytic agents [7]. Our patient experienced once potentially life-threatening bleeding; however, the current PLT count was 30 × 10^9^/l and head MRI revealed the organic cause of the hemorrhage. If the refractory patient needs therapy but all standard treatment options have failed to stably increase the PLT count, alternate therapies and combination treatments are the next step [2,5–7]. The potential benefits of combination therapies are the result of the different mechanisms of action of each drug. There are many combination therapy regimens for the treatment of refractory ITP, including corticosteroids, other immunosuppressants, TPO-RAs, and cytostatic agents [6]. According to the literature, switching to the alternate TPO-RA after the failure of the initial TPO-RA may be a favorable approach. The latest meta-analysis of switching from romiplostim to eltrombopag and *vice versa*, conducted in 2019, showed that more than 75% of patients who switched to the alternate TPO-RA maintained or achieved a response with the new treatment [18].

Avatrombopag, along with romiplostim and eltrombopag, is one of three TPO-RAs approved for the treatment of chronic primary ITP. Avatrombopag is indicated in the second-line treatment of chronic ITP and may be part of combination treatments in refractory ITP [7,19]. Due to its different structure compared with other TPO-RAs, avatrombopag can induce distinct clinical outcomes and can be used successfully in the event of the failure of the two previous drugs [20]. Romiplostim binds to the TPO receptor at the endogenous TPO binding site, whereas eltrombopag and avatrombopag act on a transmembrane part of the receptor. Unlike eltrombopag, avatrombopag is independent of intracellular iron chelation despite the same binding site [21]. The characteristics making avatrombopag a potentially beneficial option in the treatment of ITP compared with other TPO-RAs are the low potential for side effects, the absence of hepatotoxicity, the convenient oral route of administration, and the lack of interactions with food and other medications [22,23]. The efficacy of avatrombopag in the treatment of chronic ITP was proven in a randomized phase 3 study [24].

Nevertheless, the number of publications showing its real-life efficacy in treatment-resistant ITP is very limited. A clinical case was reported in which avatrombopag was administered along with fostamatinib to reduce fostamatinib-related diarrhea and then in monotherapy with the long-term maintenance of high PLT count [25]. Avatrombopag can increase PLT count with a durable effect, also for other rare reasons of thrombocytopenia as for this combined with MYH9-related disorder, successfully treated with avatrombopag, after failed treatment with eltrombopag [26]. A network meta-analysis comparing the effectiveness of TPO-RAs (romiplostim, eltrombopag, and avatrombopag) for chronic ITP in increasing PLT count showed that avatrombopag has the highest efficacy as a second-line treatment because it has the most favorable balance of benefits and acceptability [27].

Data that assess the switch of all three TPO-RAs are very limited. A small study provided real-life experience with the usage of three consecutive TPO-RAs. Six patients with chronic ITP were treated with avatrombopag after prior treatment with romiplostim and eltrombopag in either order. In the case of two patients, therapy with avatrombopag was stopped due to a lack of response. In one patient, there were also side effects (headache). In four other patients, avatrombopag was effective. However, two of these patients received corticosteroids concomitantly [14]. The authors noticed that the previous lack of response to eltrombopag may predict a lack of response to avatrombopag; however, this observation has not been confirmed in the larger retrospective observational study that evaluated the switch from a TPO-RA to avatrombopag in 44 patients with primary and secondary ITP [15]. One-third of the patients were switched to avatrombopag due to insufficient effectiveness with prior TPO-RA therapy. The platelet response (defined as PLT count ≥50 x 10^9^/l) was achieved in 86% of these patients and CR (PLT count ≥100 x 10^9^/l) in 71%. Fourteen patients were treated with all three TPO-RAs (romiplostim and eltrombopag in either order followed by avatrombopag). Seven out of 14 patients were switched to avatrombopag due to the lack of efficacy of previous TPO-RAs. Four of them achieved CR on avatrombopag. There were no differences in the response to avatrombopag depending on whether romiplostim or eltrombopag was previously ineffective [15]. This study added to the evidence that the ineffectiveness of one TPO-RA may not predict the ineffectiveness of another.

In the presented case, the limited initial period of treatment with TPO-RAs (8 weeks of romiplostim and 4 weeks of eltrombopag) was due to a lack of access to them in Poland at that time. Romiplostim and eltrombopag were donated by pharmaceutical companies. Avatrombopag administration led to sustained platelet response with PLT count of 47–52 x 10^9^/l; the patient is symptoms-free. In case of loss of response to avatrombopag, we plan to resume long-term therapy with romiplostim or eltrombopag, possibly with both simultaneously.

## Conclusion

This study provides real-life data that switching to avatrombopag may be an effective approach even in an intensively pretreated patient with chronic ITP after an inadequate response to multiple lines of treatment, including the initial failure of romiplostim and eltrombopag. This report suggests that a lack of response to one TPO-RA does not predict the ineffectiveness of another. The different efficacy in individual patients can be explained by the distinct pharmacodynamic and pharmacokinetic properties of alternate TPO-RAs. In the presented case, it should be noted that neither romiplostim nor eltrombopag caused an increase in the PLT count that was below 20 x 10^9^/l throughout the treatment period. The response was observed within 4 weeks after initiating avatrombopag. Despite the subsequent transient drop in the PLT count within the next month, there were no signs of hemorrhagic diathesis. However, a longer follow-up is needed to monitor the further response to avatrombopag.

## Acknowledgements

### Conflicts of interest

The authors report that there are no competing interests to declare.
